# Costs and Consequences of Using Interferon-γ Release Assays for the Diagnosis of Active Tuberculosis in India

**DOI:** 10.1371/journal.pone.0124525

**Published:** 2015-04-28

**Authors:** Kristen M. Little, Madhukar Pai, David W. Dowdy

**Affiliations:** 1 Department of Epidemiology, Johns Hopkins Bloomberg School of Public Health, Baltimore, Maryland, United States of America; 2 Department of Epidemiology, Biostatistics, and Occupational Health, McGill University and McGill International TB Centre, Montreal, Canada; Emory University, UNITED STATES

## Abstract

**Background:**

There is growing concern that interferon-γ release assays (IGRAs) are being used off-label for the diagnosis of active tuberculosis (TB) disease in many high-burden settings, including India, where the background prevalence of latent TB infection is high. We analyzed the costs and consequences of using IGRAs for the diagnosis of active TB in India from the perspective of the Indian TB control sector.

**Methods and Findings:**

We constructed a decision analytic model to estimate the incremental cost and effectiveness of IGRAs for the diagnosis of active TB in India. We compared a reference scenario of clinical examination and non-microbiological tests against scenarios in which clinical diagnosis was augmented by the addition of either sputum smear microscopy, IGRA, or Xpert MTB/RIF. We examined costs (in 2013 US dollars) and consequences from the perspective of the Indian healthcare sector. Relative to sputum smear microscopy, use of IGRA for active TB resulted in 23,700 (95% uncertainty range, UR: 3,800 – 38,300) additional true-positive diagnoses, but at the expense of 315,700 (95% UR: 118,300 – 388,400) additional false-positive diagnoses and an incremental cost of US$49.3 million (95% UR: $34.9 – $58.0 million) (2.9 billion Indian Rupees). Relative to Xpert MTB/RIF (including the cost of treatment for drug resistant TB), use of IGRA led to 400 additional TB cases treated (95% UR: [-8,000] – 16,200), 370,600 (95% UR: 252,200 – 441,700) more false-positive diagnoses, 70,400 (95% UR: [-7,900] – 247,200) fewer disability-adjusted life years averted, and US$14.6 million (95%UR: [-$7.2] – $28.7 million) (854 million Indian Rupees) in additional costs.

**Conclusion:**

Using IGRAs for diagnosis of active TB in a setting like India results in tremendous overtreatment of people without TB, and substantial incremental cost with little gain in health. These results support the policies by WHO and Standards for TB Care in India, which discourage the use of IGRAs for the diagnosis of active TB in India and similar settings.

## Introduction

Interferon-gamma Release Assays (IGRAs), including QuantiFERON-TB Gold In Tube (Cellestis, Carnegie, Australia) and T-SPOT.TB (Oxford Immunotec, Oxfordshire, UK), are immunological tests that are widely used to detect latent tuberculosis infection (LTBI) in high-income settings. IGRAs have higher specificity than tuberculin skin testing (TST), are less likely than TSTs to cross-react with the Bacille Calmette-Guerin (BCG) vaccine, and correlate well with *M*. *tuberculosis* exposure [1], though stronger association with progression to active disease has not been conclusively shown [2].

While IGRAs are recommended for the diagnosis of LTBI in many high-income countries, they are not recommended by the World Health Organization (WHO) as a TST replacement for LTBI diagnosis in low and middle income countries [3]. Furthermore, in no setting are IGRAs recommended for detection of active pulmonary TB [3], since IGRAs (like TST) cannot differentiate latent infection from active TB disease [4].

In high-burden settings, where 40% or more of the general population is latently infected with TB [5] and therefore likely to test IGRA-positive [6–8] (i.e., a test that is positive among all people with latent TB is likely to have a specificity of 60% or less when used for active TB), use of IGRAs to diagnose active pulmonary TB is particularly problematic. This concern equally applies to the use of tuberculin skin test (TST) for active TB. Nevertheless, in many high-burden countries, including India, there is growing concern that IGRAs (and, to a lesser extent, TST) are being used off-label, particularly in the private sector, for the diagnosis of active TB [9,10]. In India, QuantiFERON is marketed as “TB Gold,” and another IGRA (Immunoshop, Mumbai, India) as “TB Platinum,” names that provide poorly-trained healthcare providers little guidance as to their intended use. In China, domestic IGRAs are made by several companies, and publications from China suggest their use to diagnose active TB in that setting as well [11,12].

Although the use of IGRAs and TST for active TB is discouraged by the Indian Revised National TB Control Programme (RNTCP) and the Standards for TB Care in India [13], IGRAs are not banned, and there is growing concern that the use of IGRAs has increased since the ban on antibody-based serological TB tests in 2012 [14,15]. Market research conducted in 2012–2013 by the Clinton Health Access Initiative found that approximately 12% of private laboratories in India offered QuantiFERON TB Gold tests [16]. As the economic and patient consequences of this practice remain unclear, we analyzed the costs and consequences of using IGRAs for the diagnosis of active pulmonary TB in India from the perspective of the Indian TB control sector.

## Materials and Methods

### Study Design and Population

**Fig 1:**
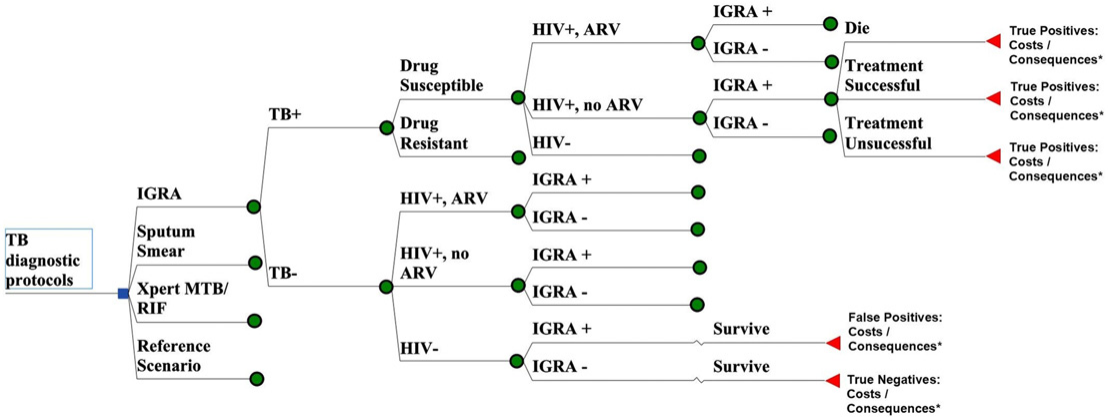
Decision Analytic Model for IGRA Testing for Active TB in India. A simplified schematic of the decision analytic model of one million TB suspects in India, with branches for the reference scenario (clinical exam only, no microbiological testing), reference scenario + testing with interferon-gamma release assays (“IGRA”), clinical exam + sputum smear microscopy (“Sputum Smear”), and clinical exam + testing with Xpert MTB/Rif with MDR treatment (“Xpert MTB/RIF”). Differential infectiousness (as denoted by smear status, in the event that a smear could be performed) is incorporated into the model, as are the reference set of tests, but these are not shown for simplicity. * “Costs” includes the cost of empiric treatment and the cost of the reference test(s), as well as the cost of the microbiological testing and TB treatment (if applicable). The “Xpert MTB/RIF” also includes the cost of MDR-TB treatment. Consequences include deaths, DALYs, secondary cases, false-positives treated, and true positives treated.

To estimate the costs and consequences of IGRAs for the diagnosis of active pulmonary TB in adults, we adapted our prior decision analysis model of serological TB tests in India [17] (Fig 1). The full model can be seen in S1 File. We took as our study population one million Indian adults in whom TB is clinically suspected, with a nationally representative prevalence of latent TB infection, human immunodeficiency virus (HIV) infection, and antiretroviral therapy access. This hypothetical study cohort is representative of adults in India presenting for TB diagnosis in settings with access to serological testing, and is intended to approximate the annual costs and outcomes among all adult patients in India receiving serological testing for suspected active TB disease. We examined costs (measured in 2013 US dollars) and effects from the perspective of the Indian healthcare sector (including both public and private sectors), with future discounting of 3% per year. Outcomes included disability-adjusted life years (DALYs) averted (without age weighting), TB cases treated, and false-positive treatments (people without active TB who are inappropriately treated for active TB).

We took our reference (base-case) scenario to consist of the existing standard of care for TB diagnosis, but without the use of any microbiological tests. Thus, this scenario would consist of clinical evaluation plus any non-microbiological tests (e.g. chest X-ray) that might be routinely performed for TB diagnosis in a typical Indian setting. We compared this reference scenario against scenarios in which this standard of care was augmented by the addition of microbiological tests, namely sputum smear microscopy, IGRA, or Xpert MTB/RIF (a WHO-endorsed, sputum-based molecular test for TB) [18]. In making this comparison, we assumed that any individual who would be treated for TB in the reference scenario would also be treated for TB after performance of a microbiological test, even if that test result was negative. (For example, someone with a strong clinical suspicion of TB, but a negative smear, IGRA, or Xpert would still be treated as having TB.) We further assumed that negative microbiological test results would not be taken as an indication to discontinue treatment among individuals started on anti-TB therapy.

In addition to smear, IGRA, and Xpert MTB/RIF, we also examined a scenario including mycobacterial culture. We do not present those findings in detail, however, as research has shown that culture results have limited impact on physician’s treatment decisions in India [19]. We included the effects of multi-drug resistant TB (MDR-TB) on each these diagnostic scenarios, assuming an MDR-TB prevalence of 2.1% among all newly diagnosed Indian adult TB cases [20], and examined the costs and consequences of diagnosing and treating MDR-TB for the Xpert arm (as, unlike smear or IGRA, Xpert is capable of diagnosing resistance to rifampin, a proxy measure for MDR-TB). Though recommended by the RNTCP in certain situations, drug-sensitivity testing (DST) is not widely available in India [21], and therefore costs associated with DST were not included in the non-Xpert MTB/RIF arms. We took as our primary outcomes the incremental costs and consequences (including DALYs, secondary TB transmissions, false positive TB cases diagnosed, and TB cases treated) of diagnosis with either IGRA or Xpert MTB/RIF, compared to the reference scenario.

### Parameters and Assumptions

Assumptions regarding the availability and turnaround time for sputum smear microscopy are as previously reported [17], and include a one week turnaround time for sputum smear microscopy with a loss to follow-up during this interval of 15% [22]. We assumed that IGRAs would be performed by private laboratories via send-out testing, with a 1-week turnaround time and a loss-to-follow-up rate similar to that of sputum smear microscopy. To be conservative, Xpert MTB/RIF was assumed to have a delay between testing and treatment initiation of 7 days [23], and the same loss to follow-up as with IGRAs or sputum smear microscopy. Diagnostic testing cost estimates were based on published data, inflated to the year 2013 using the Indian GDP deflator [24], followed by conversion into US dollars at the 2013 exchange rate [25], where applicable.

We estimated the cost of sputum smear microscopy to be US$3 (range: US$1–5, for two smears) for each TB suspect tested [26]. Based on an internal survey (unpublished) of private laboratories [27], we estimated that IGRAs would cost US$30 (range: US$20–50) per test, while Xpert MTB/RIF was assumed to cost US$25 per test (range: US$20–57) [28,29]. After analysis overall costs were converted into 2013 Indian Rupees (INR) from 2013 US dollars using historical exchange rates [25], and cost outcomes are presented in both currencies.

Test accuracy estimates were obtained from the published literature, including meta-analyses where available. IGRA accuracy values were based on the QuantiFERON-TB Gold test, and obtained from studies performed in low- and middle-income country settings. While the T-SPOT.TB IGRA is unavailable in India, QuantiFERON-TB Gold In-Tube is available and commonly utilized, especially in the private sector [30]. For our analysis we used an IGRA sensitivity of 0.84 [4] (range: 0.56–0.96). For the purposes of illustrating the sensitivity of our model to a wider interval of possible sensitivities of IGRA tests, we used a wider range for sensitivity analysis around this variable than the published pooled confidence interval [4]. We used an IGRA specificity (for active TB, in individuals with suspected TB in low and middle income countries) of 0.52 (range: 0.40–0.79) [4]. Additional parameter values are given in Table 1.

**Table 1:** Estimates for Model Parameters.

Parameter	Base Value	Range for Sensitivity Analysis	Source
**TB Dynamics**			
Probability of death, untreated smear-positive TB¹	0.70	0.50–0.95	[37]
Probability of death, untreated smear-negative TB	0.20	0.15–0.25	[37]
Secondary TB infections per year, smear-positive TB	10	8–12	[38]
Relative infectiousness of smear-negative TB	0.22	0.16–0.28	[39]
Fraction of new TB cases that are smear-positive	0.53	0.40–0.66	[37]
**Characteristics of TB diagnosis**			
Prevalence of active TB among persons with suspected active TB	0.14	0.11–0.18	[40]
Sensitivity for TB			
Clinician diagnosis²	0.53	0.40–0.67	[31]
Sputum smear microscopy	0.53	0.34–0.65	[41]
IGRA³ (QuantiFERON-TB Gold)	0.84	0.56–0.96	[4]
Xpert MTB/RIF^4^ (smear-positive TB)	0.98	0.97–0.99	[18]
Xpert MTB/RIF (smear-negative TB)	0.67	0.58–0.74	[18]
Specificity for active TB^5^			
Clinician diagnosis	0.94	0.75–1.00	[42]
Sputum smear microscopy (two smears)	0.97	0.75–1.00	[43]
Xpert MTB/RIF	0.98	0.97–0.99	[18]
IGRA (QuantiFERON-TB Gold)	0.52	0.41–0.62	[4]
Xpert MTB/RIF Rif resistance			
Sensitivity	0.94	0.87–0.97	[18]
Specificity	0.98	0.97–0.99	[18]
Time to TB diagnosis (days)			
Sputum smear microscopy	7	2.92–14.05	[44,45]
GeneXpert MTB/RIF	7	0.50–14.05	
IGRA (QuantiFERON-TB Gold)	7	2.92–14.05	
Loss to follow-up			
Sputum smear microscopy	0.15	0.11–0.19	[46,47]
Xpert MTB/RIF	0.15	0.11–0.19	
IGRA (QuantiFERON-TB Gold)	0.15	0.11–0.19	[17]
**Characteristics of TB treatment**			
Proportion of treated TB patients who die	0.045	0.033–0.056	[31]
Proportion of treated HIV^6^/TB patients who die	0.090	0.068–0.114	[31]
Proportion of treated TB patients infections at 1 year	0.045	0.033–0.056	[31]
**HIV/TB**			
HIV prevalence, general population	0.3%	0.225–0.4%	[48]
HIV prevalence, patients with TB	5.3%	4.0–6.6%	[31]
Proportion of HIV-infected patients with ART^7^ access	0.10	0.075–0.125	[48]
**Costs and effectiveness, US Dollars (2013)**			
Unit cost, independent laboratory			
Sputum smear microscopy (two smears)	$3.00	$1-$5	[24,49]
Xpert MTB/RIF	$25	$20-$57	[50]
IGRA (QuantiFERON-TB Gold)	$30	$15-$50	[27,30]
Mean cost of treating one case of drug-susceptible TB	$66.00	$50-$75	[31]
Mean cost of treating one case of MDR^8^ TB	$2600	$500-$5500	[31]
DALY^9^ weights			
Active TB	0.264	0.198–0.330	[51]
TB treatment	0.132	0.099–0.165	[17]
Life expectancy after TB cure (years)	40	30–50	[52,53]

^1^ TB, Tuberculosis

^2^ In the absence of any TB-specific microbiological test

^3^ IGRA, Interferon-Gamma Release Assay

^4^ MTB/RIF, *Mycobacterium tuberculosis* and Rifampin Resistance Testing

^5^ Excludes studies not performed in developing countries

^6^ HIV, Human Immunodeficiency Virus

^7^ ART, antiretroviral therapy

^8^ MDR, Multi-drug resistant

^9^ DALY, Disability-Adjusted Life Year

### Sensitivity Analysis

We performed one-way sensitivity analyses on all model parameters, taking as the primary outcome the incremental costs and consequences of IGRA compared to the reference standard. Ranges for each model parameter are listed in Table 1. We performed additional two-way sensitivity analyses on those parameters with the greatest effects on model outcomes. Where data were lacking in regards to an appropriate range for the sensitivity analysis for a given parameter, we varied each parameter value by +/- 25% beyond its base value.

Finally, we performed a probabilistic sensitivity analysis (PSA) to incorporate uncertainty in all model parameters. In this analysis, we simultaneously varied all model parameters using Monte Carlo simulation (10,000 simulations), assuming that all parameters followed beta distributions with a mode equal to the most likely value (in Table 1) and an alpha (shape) parameter of four, with two exceptions. First, since it was unlikely that accuracy values (sensitivity and specificity) would approach zero, we varied these parameters using triangular distributions with minimum and maximum values based on the upper and lower estimates derived from systematic reviews where possible, and other published literature values where not. Second, some values (e.g., costs) do not have a natural upper bound; for these parameters, we assumed a gamma distribution with a mode at the most likely value and an alpha parameter of 100. The full model used for the PSA can be seen in S2 File. All analyses were conducted using TreeAge Pro 2013 (TreeAge Software, Inc., Williamstown, MA, USA).

## Results

**Fig 2:**
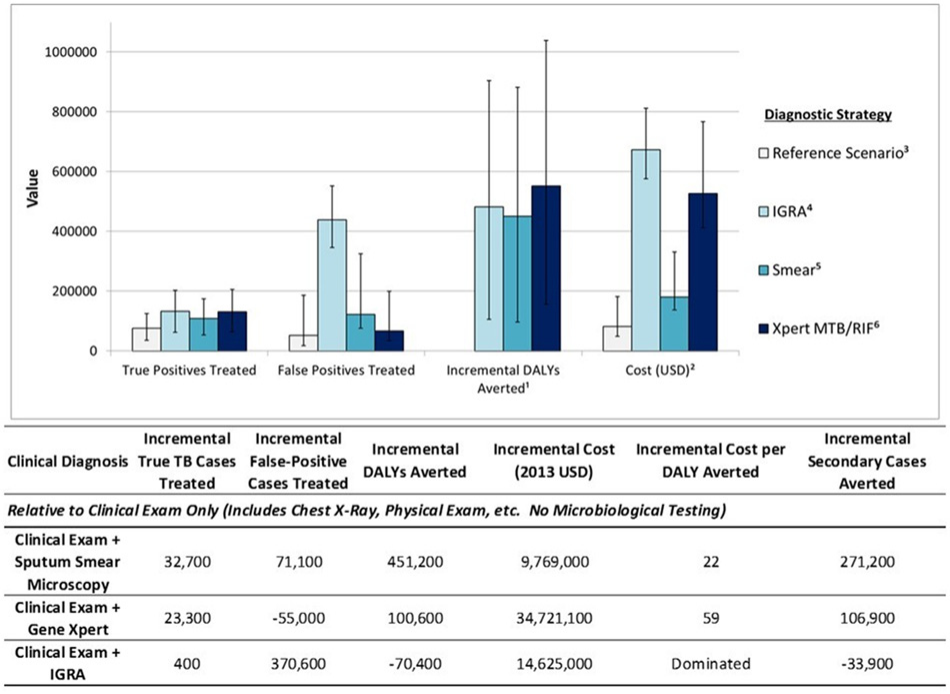
Economic and epidemiological outcomes among 1 million adults with TB symptoms in India. Model outcomes, including true positive TB cases treated, false positive cases treated, incremental DALYs averted, and costs (in 2013 US dollars) are presented below for each diagnostic strategy evaluated in the decision-analytic model. ^1^ Compared to the reference scenario. ^2^ Costs are in 2013 US Dollars/100. ^3^ The reference scenario consists of clinical examination only, including chest X-Rays, physical exam, etc. No microbiological testing is considered in the reference scenario. ^4^ Interferon Gamma Release Assays. ^5^ Sputum smear microscopy. ^6^ Gene Xpert testing, including costs for treatment of drug-resistant tuberculosis

Our hypothetical cohort of one million people with TB symptoms included 143,000 individuals with active TB. When used alone, we estimated that clinical examination and reference scenario tests (such as X-ray) would detect 75,700 (53%) (95% Uncertainty Range, UR: 36,000–124,900) of these patients (Fig 2). Addition of sputum smear microscopy to this algorithm increased the overall yield to 108,400 (76%) (95% UR: 53,800–174,300) (Fig 2). Use of Xpert MTB/RIF instead of smear for all members of the cohort identified an additional 23,300 cases relative to smear (yield: 131,700, 92%) (95% UR: 64,100–206,100), while also identifying 55,000 fewer false-positive cases than smear microscopy (95% UR: 5,100–198,800). Use of mycobacterial culture methods (under the additional assumption that culture results could largely be translated into treatment decisions, with 10% additional loss to follow-up) resulted in qualitatively similar outcomes as those observed in the Xpert MTB/RIF arm (data not shown).

Use of IGRA rather than smear had a similar yield of true-positive diagnoses as Xpert (132,100 cases, 93%) (95% UR: 62,800–203,000), but at the expense of 438,200 false-positive diagnoses (95% UR: 345,600–552,400)—3.3 false-positives diagnosed for every true-positive—and 315,700 (95% UR: 118,300–388,400) more false-positives than sputum smear microscopy.

The cost of IGRA-based diagnosis, from the Indian healthcare perspective, was also high. For every million individuals tested by IGRA rather than sputum smear microscopy, the incremental cost of TB diagnosis and treatment was US$49.4 million ($67.3 million with IGRA vs. $18.0 million with sputum smear microscopy) (95% UR: $34.9–58.0 million) (Fig 2). The US$49 million (2.9 billion Indian Rupees, INR) in incremental costs reflected approximately US$27 million (1.6 billion INR) in additional diagnostic test costs, US$2 million (117 million INR) in the treatment of additional true-positive cases identified with IGRAs, and nearly US$21 million (1.2 billion INR) for the treatment of false-positive cases—people without active TB who nevertheless received six months of anti-TB drugs.

Similarly, although the cost of diagnosis with IGRA and Xpert MTB/RIF was similar, the total healthcare cost of IGRA was nearly US$15 million greater (95% UR: $-7.2–28.7 million) (854 million INR) than Xpert (US$67.3 million vs. $52.7 million) (3.9 billion vs. 3.1 billion INR), due to the large numbers of false-positive diagnoses triggering inappropriate treatment (Fig 2). Incorporating the possibility of MDR treatment for IGRA-diagnosed false positive cases would raise the total costs of the IGRA scenario even more. As a result of the poor specificity of IGRA, we estimated that Xpert MTB/RIF would avert more DALYs, and at lower cost, than IGRAs (as used for diagnosis of active TB), even after including the costs of treating drug-resistant TB in the Xpert MTB/RIF scenario. Compared to Xpert MTB/RIF, the use of IGRAs diagnosed only 400 additional true TB cases (95% UR: -8,000–16,200), and made 370,600 more false-positive diagnoses (95% UR: 252,200–441,700). Relative to sputum smear microscopy alone, Xpert MTB/RIF cost US$345 (20,200 INR) per DALY averted, similar to prior analyses [28], while the IGRA strategy was dominated by the Xpert strategy.

**Fig 3:**
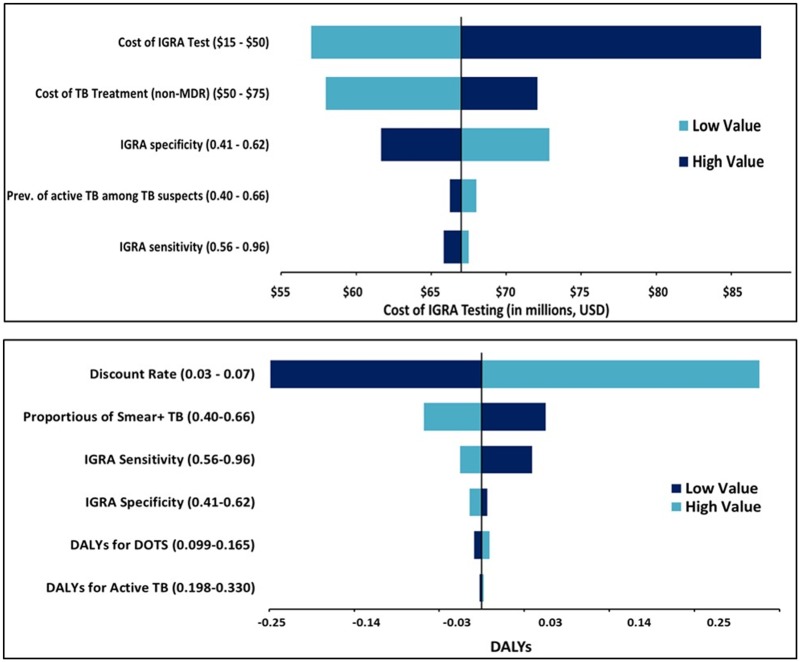
One-Way and Two-Way Sensitivity Analyses on Parameters Affecting Cost and DALYs Averted. Top) Tornado diagram examining model parameters with the largest impact on the cost of the IGRA testing strategy. Bottom) Tornado diagram examing model parameters with the highest impact on DALYs averted by IGRA testing.

In one-way threshold analyses based on the pre-specified ranges of all variables in the model (Fig 3), the scenario using Xpert MTB/RIF averted more DALYs than IGRAs at IGRA sensitivities of 95.7% or less, very near the upper bound of 96%. When considering the cost of MDR treatment based on Xpert results, IGRA-based algorithms were more costly than Xpert-based ones unless the unit cost of Xpert exceeded US$39 (2,280 INR). Xpert MTB/RIF remained less costly than IGRA testing until the average cost of MDR treatment exceeded US$5100 (298,000 INR) per person, near the highest average cost of MDR treatment in India from 2008–2013 (US$5500) [31] (322,000 INR). In other scenarios tested, Xpert MTB/RIF (including MDR treatment) was both less expensive and more effective than IGRA testing.

**Fig 4:**
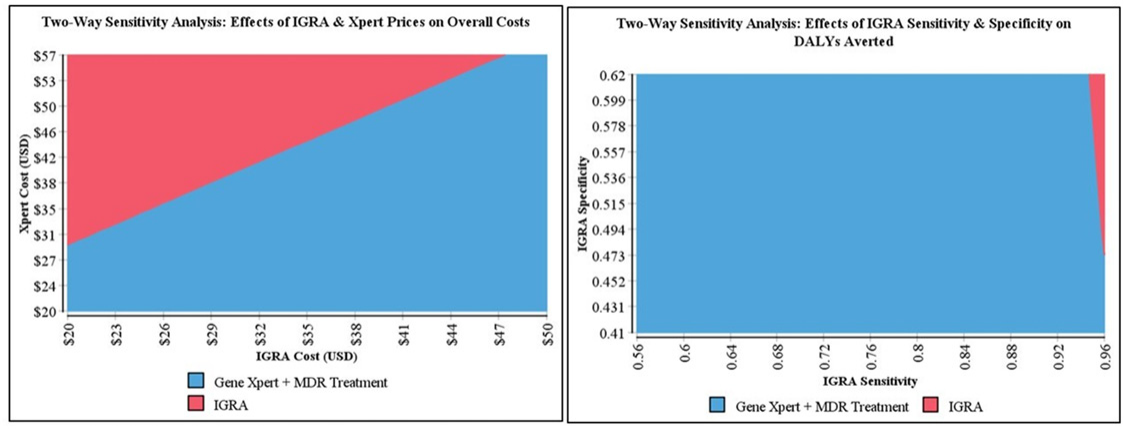
Select Two-Way Sensitivity Analyses. Left) Two-way sensitivity analysis of the effects of changes in IGRA sensitivity and specificity parameters within pre-specified ranges on DALYs averted, demostrating the most effective diagnostic strategy, irrespective of cost. Right) Two-way sensitivity analysis of the effects of changes in IGRA and Xpert MTB/Rif prices within pre-specified ranges on the cost of each diagnostic approach, demostrating the least costly diagnostic strategy, irrespective of effectiveness.

In two-way sensitivity analyses, even with a specificity of 62% (the upper bound of IGRA specificities tested), IGRAs only averted more DALYs than Xpert MTB/RIF in situations in which the IGRA sensitivity was over 94% (Fig 4). In terms of overall costs, two-way sensitivity analysis found that even if IGRAs cost only US$20 (1,170 INR) per test (the lowest bound of IGRA costs tested), algorithms using Xpert MTB/RIF would still be less costly below a unit price of US$29 (1,700 INR) per Xpert test.

In probabilistic sensitivity analysis, IGRA was more costly than Xpert (without MDR treatment) in 100% of the simulations, and more costly than Xpert (including MDR treatment) in 89% of simulations. Xpert averted more DALYs than IGRAs in 96% of simulations, and sputum smear microscopy averted more DALYs than IGRAs in 48%.

## Discussion

More than 2 million individuals are diagnosed with active TB every year in India, leading to approximately 300,000 TB-attributed deaths [20]. Although diagnosis and treatment of LTBI is a valid TB control strategy in specific high-risk groups [32], given current resource constraints, national TB programs, including the Indian RNTCP, prioritize treatment of active TB over LTBI. A ban on antibody-based serodiagnostic tests and a provider preference for blood-based rather than sputum-based diagnostics [33] has expanded the market for IGRAs. Indeed, Indian physicians rarely treat LTBI in routine clinical practice [34], but tests for LTBI, including not only IGRAs but also TST, are commonly used [15]. Our analysis shows the likely adverse economic consequences to the healthcare system when IGRAs are utilized to diagnose active TB in areas with high background prevalence of LTBI. Given the growing popularity of IGRAs in other countries such as China, where evidence suggests the tests are also being used to diagnose active TB disease, these economic and healthcare system concerns likely extend to settings beyond India as well [11,12].

Based on the prevalence of LTBI in India’s adult population [5], IGRAs—even with perfect specificity for their designed indication (diagnosis of LTBI)—would be positive in more than 40% of all people without active TB, meaning that for every individual appropriately diagnosed with TB using IGRAs, about 3 individuals would be falsely diagnosed and subjected to six months of treatment. Treating these individuals for active TB—even in a country with TB treatment costs among the lowest in the world—would cost US$36.1 million (2.1 billion INR) for every 1 million people with TB symptoms. By comparison, appropriate treatment for all individuals in the cohort with MDR-TB—an expense that has, in the past, been argued to be unsupportable by the Indian TB control program—would cost US$7.6 million (440 million INR), and even the use of commercial serology—now banned due to its poor performance—would cost less (US$31.7 million, 1.9 billion INR) and make fewer false-positive diagnoses (75,500 vs. 438,200) than IGRAs [17]. While we did not explicitly model the use of TST for active TB diagnosis in this analysis, these adverse consequences would be similar (though the cost of testing might be less), as neither IGRAs nor TST can distinguish LTBI from active TB disease.

While IGRAs are commercially available and commonly used in the Indian private healthcare sector, research has revealed substantial confusion and inconsistency in the interpretation of IGRAs on the part of healthcare practitioners [30]. A recent survey of Indian pulmonologists, ophthalmologists, and rheumatologists found that 91.9% of those surveyed reported using IGRAs “routinely” or “sometimes” in their practices. This same survey found that, despite the widespread use of IGRAs among those surveyed, more than 80% of those surveyed believed that the test was able to differentiate between latent and active TB, and only 13% believed that the test was mainly intended for diagnosing latent, and not active, TB infection. The main barrier to the use of IGRAs among those surveyed was the higher cost of the test [30]. As this analysis demonstrates, however, the major cost to the health system of IGRA testing for active TB results from the treatment of the substantial number of false-positive TB patients identified by IGRAs.

In comparison, Xpert MTB/RIF identified a similar number of true positive TB cases as IGRAs (131,700 vs. 132,100), while making considerably fewer false-positive diagnoses (67,500 vs. 438,200). These additional false positive diagnoses by IGRAs represent a misallocation of financial and human-resources, as well as added health risks for individuals wrongly undergoing treatment for active TB. In addition to increased specificity and same-time drug sensitivity testing, Xpert MTB/RIF can provide TB results in as little as an hour, making same-day diagnosis and treatment initiation a possibility. Another significant advantage of Xpert MTB/RIF is the ability to rapidly perform drug susceptibility testing. While accounting for the expense associated with MDR treatment for drug resistant TB cases naturally increases the overall cost of the Xpert MTB/RIF scenario, appropriate, timely treatment of drug-resistant TB is likely cost-saving in the long term through the prevention of secondary MDR TB transmission and a reduction in MDR TB-associated morbidity and mortality. A recent model has explored the optimum implementation strategy to maximize the impact of Xpert MTB/RIF in India [35], and the price of this test has been reduced by nearly 50% in the private sector via an Initiative to Promote Affordable, Quality TB Tests (IPAQT), supported by the Clinton Health Access Initiative [36].

Our analysis has important limitations. By adopting a healthcare perspective, and thereby ignoring the costs of TB treatment borne by patients and their families, or the erosion of trust in the healthcare system following misdiagnosis, we may underestimate the societal cost of false-positive TB diagnosis. Thus, our estimates of cost-effectiveness may actually be biased in favor of IGRAs compared to a societal analysis. Additionally, we used published data rather than empirical estimates for our parameter values, which often vary considerably and may not be fully generalizable to an Indian context. Nevertheless, our findings were robust to wide parameter variation in sensitivity analysis, and our estimates likely underestimate the cost of false-positive TB diagnosis in healthcare systems where treatment of TB is more costly than in India. Finally, we adopted a simplified model structure that, for example, assumed most people who tested positive for TB completed a full course of treatment, and that providers used IGRA results to diagnose active disease, rather than LTBI. This model therefore does not fully capture the complex dynamics of the system in which TB diagnosis is actually performed.

Improved diagnostics are a critical tool to control TB worldwide, and IGRAs have an important role to play in the diagnosis of LTBI, particularly in low—risk settings. The 2014 WHO guideline on management of LTBI suggests that either TST or an IGRA can be used to test for LTBI in high-income and upper middle-income countries with estimated TB incidence less than 100 per 100 000 [32]. The guideline recommends that IGRA should not replace TST in low-income and other middle-income countries [32]. However, in the setting of severely constrained resources for TB control, it is also important to deploy existing diagnostic tests in a manner that is both responsible and cost-effective. In such settings, tests for LTBI are likely to be most useful and cost-effective if restricted to select high-risk groups (e.g. child contacts of active TB cases, immunosuppressed populations) in whom test results might feasibly be linked to LTBI treatment, and for whom such treatment offers the greatest clinical benefit [32]. This analysis quantitatively demonstrates that, due to the potential for massive misdiagnosis of individuals with LTBI as active disease, the use of IGRAs to diagnose active TB in high-burden settings is likely to result in tremendous wastage of vital resources, and at substantial loss of health, even relative to insensitive tools such as sputum smear microscopy. The Standards for TB Care in India explicitly discourage the use of IGRAs and TST for active TB diagnosis [13], but greater efforts are necessary to raise awareness about this recommendation among Indian clinicians and laboratorians, and to redirect TB control resources to microbiological tests with known validity for diagnosing active TB.

## Supporting Information

S1 File TreeAge Decision-Analytic Model for IGRA Cost-Effectiveness Analysis in India. This ThreeAge .trex file contains the full model and all parameters used for this analysis.
(ZIP)

S2 File TreeAge Probabilistic Sensitivity Analysis for IGRA Cost-Effectiveness Analysis in India. This ThreeAge .trex file contains the full model and all parameters used in the probabilistic sensitivity analysis.
(ZIP)
